# To the Editors of the Medical and Physical Journal

**Published:** 1801-04

**Authors:** W. Milburne

**Affiliations:** Stockton on Tees


### To the Editors of the Medical and Physical Journal.

GENTLEMEN,

I Take the liberty to communicate the following remarkable cafe for your confideration; I do not know that it will throw any light on the healing art, its fingularity only prompted me to the detail, which probably you may think worthy a place in your valuable publication. I am, &c.

*Stockton on Tees, Feb.* 26, 1801. W. MILBURNE.

---

Jofeph Rodgers, aged 50, about four miles from Stockton, on the 12th of December 1800, cut his throat with a razor in the moft determined manner. He had firft paffed the razor over the larynx and pharynx, dividing thofe from the upper parts: he then made another incifion about three inches below, through the trachea and œfophagus; not finding this effectual, he diffected out the divided parts, with the os hyoides, epiglottis, and attendant mufcles and integuments, &c. The inftrument had not been keen, as it appeared from the cartilaginouspart of the trachea to have been cut through at repeated ftrokes. He was found foon after in a garden, fitting on his knees, his body leaning forward over a piece of timber, (the razor and parts diffected, with a quantity of blood, lying at his feet) to all appearance dead. Cloths were immediately applied round his neck to cover the frightful wound, and he was conveyed to a houfe; in a few minutes he began to fhew figns of life, and in half an hour, walked up one pair of ftairs unfupported to bed; I then was fent for, found him perfectly collected, and from figns, forry for what he had done. On removing the cloth to examine the wound, the preffure of external air on the lungs threatened inftant fuffocation, which being immediately covered, he feemed perfectly at eafe, in which ftate he continued to the eighth day, and expired completely exhaufted.

P. S. If it is requefted, I fhall fend the parts wounded, which are preferved in fpirits.

